# LncRNA ZEB1-AS1/miR-1224-5p / MAP4K4 axis regulates mitochondria-mediated HeLa cell apoptosis in persistent *Chlamydia trachomatis* infection

**DOI:** 10.1080/21505594.2022.2044666

**Published:** 2022-03-10

**Authors:** Fangzhen Luo, Yating Wen, Lanhua Zhao, Shengmei Su, Wenbo Lei, Lili Chen, Chaoqun Chen, Qiulin Huang, Zhongyu Li

**Affiliations:** a Institute of Pathogenic Biology, Hengyang Medical School, Hunan Provincial Key Laboratory for Special Pathogens Prevention and Control, University of South China, Hengyang, P. R. China; b College of Medical Technology, Hunan Polytechnic of Environment and Biology, Hengyang, P. R. China; c The First Affiliated Hospital, Hengyang Medical School, University of South China, Hengyang, P. R. China

**Keywords:** *Chlamydia trachomatis*, lncRNA ZEB1-AS1, miR-1224-5p, MAP4K4, antiapoptosis, persistent infection

## Abstract

Persistent infection of *Chlamydia trachomatis* is thought to be responsible for the debilitating sequelae of blinding trachoma and infertility. Inhibition of host cell apoptosis is a persistent *C. trachomatis* infection mechanism. ZEB1-AS1 is a long non-coding RNA (lncRNA), which was up-regulated in persistent *C. trachomatis* infection in our previous work. In this study, we investigated the role of ZEB1-AS1 in persistent infection and the potential mechanisms. The results showed that ZEB1-AS1 was involved in the regulation of apoptosis, and targeted silencing of ZEB1-AS1 could increase the apoptosis rate of persistently infected cells. Mechanically, interference ZEB1-AS1 caused an apparent down-regulation of the Bcl-2/Bax ratio and the repression of the mitochondrial membrane potential with the remarkable release of cytochrome c, resulting in the significant elevation level of caspase-3 activation. Meanwhile, the luciferase reporter assay confirmed that ZEB1-AS1 acted as a sponge for miR-1224-5p to target MAP4K4. The regulatory effect of miR-1224-5p/MAP4K4 on persistent infection-induced antiapoptosis was regulated by ZEB1-AS1. In addition, ZEB1-AS1 inhibited the apoptosis of *Chlamydia*-infected cells by activating the MAPK/ERK pathway. In conclusion, we found a new molecular mechanism that the ZEB1-AS1/miR-1224-5p/MAP4K4 axis contributes to apoptosis resistance in persistent *C. trachomatis* infection. This work may help understand the pathogenic mechanisms of persistent *C. trachomatis* infection and reveal a potential therapeutic strategy for its treatment.

## GRAPHICAL ABSTRACT

**Figure:**
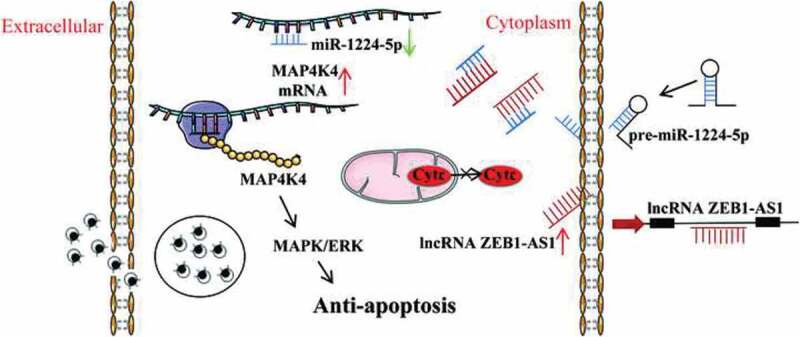

## Introduction

C.*trachomatis* is an obligate intracellular parasitic pathogen. Its persistent infection mediates an irreversible process of inflammatory damage [1]. The persistent form of this intracellular bacteria in the infected mucosa and epithelial cells is thought to initiate the pathogenic events leading to urethritis, pelvic inflammatory, ectopic pregnancy, oocyte sterility, and so on [2,3]. Studying the pathogenic mechanism of persistent *C. trachomatis* infection is very important for controlling associated diseases [4].

Apoptosis is an important defense mechanism against intracellular pathogens [5]. *C. trachomatis* has evolved many evasive strategies to promote its survival by regulating infected host cells’ programmed cell death pathways [6–8]. Preceding reports have indicated that *C. trachomatis* induces host cells antiapoptosis involving host protein [9–11]. However, host protein expression is usually regulated by non-coding RNAs (ncRNAs) [12]. Accordingly, whether ncRNAs are associated with persistent *C. trachomatis* infection-induced antiapoptosis and the specific molecular mechanism should be further addressed.

In recent years, lncRNA has been related to regulating various biological processes of the host by intracellular pathogens, such as *Rickettsia conorii* [13], *Mycobacterium tuberculosis* [14, *]Helicobacter pylori* [15], *Listeria monocytogenes* [16], and especially virus [17,18]. During Kaposi’s sarcoma-associated herpesvirus (KSHV) infection, researchers identified a noncoding polyadenylated nuclear RNA that is essential for viral replication and antiviral reaction [19]. In 2013, Humphreys et al. showed a high number of Chlamydia-specific transcripts at 24 hpi that suggest more interaction between *C. trachomatis* and cells [20]. Now, the role of lncRNAs in the host response to *C. trachomatis* remains a mystery. Our team previously identified the up-regulated expression of ZEB1-AS1 in HeLa cells with persistent *C. trachomatis* infection through lncRNA microarray. In the previous study, ZEB1-AS1 was proposed as an essential molecule in tumor proliferation and apoptosis [21]. However, the molecular function and mechanism of ZEB1-AS1 in persistent infection are unknown.

In this work, we firstly sifted microarray data to identify the differentially up-regulated ZEB1-AS1 and mRNA MAP4K4 in persistent *C. trachomatis* infection. Secondly, the interacting molecules and potential signaling pathways regulated by ZEB1-AS1 were determined using bioinformatics analysis, and ZEB1-AS1 was speculated to be a key molecule in the process of host cell apoptosis. Finally, molecular biological experiments were used to verify the interaction and regulation among ZEB1-AS1, miR-1224-5p, and MAP4K4 and characterize the role of ZEB1-AS1 in apoptosis resistance induced by MAPK/ERK pathway activation in persistent *C. trachomatis* infection. These findings provide new insights into the molecular mechanisms underlying persistent *C. trachomatis* infection and ultimately improve the treatment of persistent *C. trachomatis* infection.

## Materials and methods

### C. trachomatisand cell culture

HeLa 229 cells were cultured in DMEM (Dulbecco’s Modified Eagle Medium; Gibco) containing 10% (v/v) fetal bovine serum (FBS; Evergreen) at 37°C in an incubator with 5% CO_2_. The standard strain of *C. trachomatis* serovar E was stored in our laboratory.

### Persistent C. trachomatis infection

HeLa 229 cells were inoculated on 6-well plates or 24-well plates at 37°C with 5% CO_2_ overnight. The cells were pretreated with DEAE (Diethylaminoethyl dextran gel, 30 μg/ml) for 15 min, and the DEAE solution was discarded. The infection solution (MOI = 1) was quickly added to the cell culture plate after washing with DMEM, followed by 300×*g* centrifugation for 1 h to assist infection. Finally, the infection solution was replaced with a fresh medium supplemented with 75 U/mL recombinant human IFN-γ (Solarbio) for an additional 24 h. The presence of *C. trachomatis* was detected by transmission electron microscopy.

### Quantitative real-time PCR

Total RNA was extracted using Trizol reagent (Invitrogen) following reagent specifications. Total RNA and reverse transcription reagent (Tiangen) were prepared to obtain cDNA. The expression of ZEB1-AS1 and MAP4K4 were calculated by the 2^−ΔΔCt^ method using SuperReal PreMix Plus kit (Tiangen), and *18S rRNA* acted as an internal control. The expression of miR-1224-5p was calculated by the 2^−ΔΔCt^ method using miRcute miRNA kit (Tiangen), and U6 served as an internal control. qRT-PCR was performed on a LightCycle 96 apparatus (Roche, Basel, Switzerland). The amplification procedure was a three-step process consisting of 40 cycles, and three parallel replicates were performed for all results. The specific qRT-PCR primer sequences were listed in Supplementary Table 1.

### Mitochondrial membrane potential (MMP) analysis

MMP was observed in HeLa 229 cells stained with JC-1 (Beyotime) by fluorescence microscope. The carbonyl cyanide 3-chlorophenylhydrazone (CCCP) provided in the kit was added to the cell culture medium for 20 min as a positive control. 1 mL of JC-1 staining working solution was added and mixed thoroughly. The plates were incubated in a cell incubator at 37°C for 20 min. After incubation, green fluorescence was observed under the fluorescence microscope, suggesting that the cells might be in the early stage of apoptosis with MMP decline. In contrast, red fluorescence indicated that the MMP was relatively normal.

### Immunofluorescence analysis

After discarding the medium in the culture plates, the adherent cells were fixed with 4% paraformaldehyde for 30 min. Then the cells were permeated with .1% Triton X-100 for 10 min after washing with PBS. Following Triton X-100 was discarded, PBS was washed twice and blocked with DMEM medium containing 10% FBS for 1 h. Subsequently, washing off the blocking solution, the primary antibody (rabbit anti-cytochrome c; Abcam) was diluted in DMEM medium at 1:200 and added to the culture plate for 1 h. Subsequently, Cy3-labeled goat anti-rabbit fluorescent secondary antibody (1:200; Proteintech) and Hoechst 33258 (1:1000; MedChemExpress) were added to the culture plates under dark conditions at 37°C for 1 h. Finally, they were observed and photographed under a fluorescence microscope.

### RNA Fluorescent in situ hybridization (RNA-FISH)

ZEB1-AS1 probe-1 and probe-2 were synthesized by General Bio, Inc. The specific sequences are listed in Supplementary Table 1. ZEB1-AS1 probes were labeled with 5' Cy3 fluorescent dye. After the cells were fixed and permeated, the probes hybridization solution was added. Then the cells were washed, and the nuclei were stained using DAPI (Beyotime). An inverted fluorescence microscope (ECLIPSE TE2000–5) was used for fluorescence detection.

### Bioinformatics analysis

In this study, MeV V4.9.0 software was used to cluster the data and obtain the heatmap, which showed the differential expression data of ZEB1-AS1 and MAP4K4 in more detail. LncBase v.2, miRDB, and starbase 3.0 sites were applied to predict the possible targets of ZEB1-AS1, miR-1224-5p, and MAP4K4, respectively. Then YM500v2 site was used to predict the abundance of miRNA in tissues. Starbase was applied to forecast binding sites of specific miR-1224-5p interacting with ZEB1-AS1 and MAP4K4. Furthermore, ClueGo Plugin analyzed the ZEB1-AS1 targeted mRNAs and enrichment function visualized by Cytoscape 3.6.1 (https://cytoscape.org/).

### Dual-Luciferase reporter gene

According to the fragments of ZEB1-AS1 and MAP4K4 binding to miR-1224-5p predicted by ENCORI, we constructed target sequences wild-type and mutant psicheck2 vectors (Sangon Biotech). First, the target sequences were amplified, and then the PCR products and psicheck2 vectors were subjected to *Not*I and *Xho*I double enzymes digestion. After purification and recovery, the target gene sequences (Supplementary Table 2) and psicheck2 vector nucleic acid fragments were obtained and connected. The recombinant plasmids linked with target sequences were transformed into competent cells for propagation, and the recombinant plasmids were extracted and identified. ZEB1-AS1 wild-type (WT), ZEB1-AS1 mutant-type (MUT), MAP4K4 WT, and MAP4K4 MUT were generated. Subsequently, the above vectors (.4 μg) and miR-1224-5p mimic (50 nM) were co-transfected with lipofectamine 3000 (Invitrogen). After transfection for 48 h, according to the instructions, the binding sites were validated using a dual-luciferase reporter assay kit (Promega, USA).

### RNA interference

Small-interfering RNAs (siRNAs) targeting ZEB1-AS1 and MAP4K4 (siZEB1-AS1, 50 nM; siMAP4K4, 50 nM), scrambled siRNA (siNC, 50 nM, as a control group), miR-1224-5p mimic (50 nM), miR-1224-5p inhibitor (200 nM) and corresponding negative controls (NC mimic, 50 nM and NC inhibitor, 200 nM) were designed and synthesized from Sangon Biotech. According to the transfection instructions, the adherent cells were transfected with lipofectamine 3000 (Invitrogen) when the cell density was about 50%. We performed the following tests after 24 h transfection. Western blot and qRT-PCR were used to detect the expression level of the transfected samples and related functional tests. The detailed siRNAs sequences are listed in Supplementary Table 1.

### Hoechst 33258 staining

HeLa cells were treated with siNC or siRNA for 24 h and then were followed by persistent infection for 24 h. The uninfected groups were treated with fresh medium for 24 h. Then fixation, penetration, and sealing were performed. Finally, Hoechst 33258 (1:1000) was added to avoid light staining for 30 min. After washing with PBS 5 times, apoptotic bodies were observed and counted under the fluorescence microscope. The apoptotic bodies were dense and highly stained. We randomly selected five fields (200×) from each well in each sample and calculated the total number of cells and apoptotic bodies in the visual field. The apoptotic rate of each well was calculated according to the formula (apoptotic rate = apoptotic number/total cell number × 100%).

### Flow cytometry (FCM) analysis

The apoptosis rate of all samples was detected using the FITC-Annexin V and propidium iodide (PI) kit (US Everbright). The sample cells were digested and collected with trypsin, centrifuged, and washed, then 5 μL FITC-Annexin V and 10 μL PI working solution were added to each group. After incubation at room temperature for 10 min, we added 400 μL PBS and detected each group within 1 h (FACS Calibur, BD, USA).

### Western blot analysis

The samples were added 100 μL RIPA (Solarbio) cell lysate containing 1 mM PMSF (Solarbio) and incubated on ice for 30 min. Cells were scraped evenly, and the lysate was collected into an EP tube. After centrifugation at 4000 ×*g* for 10 min, the supernatant was obtained, and the protein concentration was determined by the BCA method (Beyotime). Then SDS-PAGE gel electrophoresis was performed after adding loading buffer into the supernatants. The following proteins were transferred to a PVDF membrane (.22 μm; Millipore, Billerica, MA, USA). The primary antibody (anti-Bax, anti-Cleaved Caspase-3, anti-β-actin, anti-GAPDH, Abcam; anti-MAP4K4, Immunoway; anti-Bcl-2, anti-t-ERK, anti-p-ERK, anti-t-JNK, anti-p-JNK, anti-t-p38, anti-p-p38, CST; anti-pORF5 [22,23], MAb clone 2H4) was incubated overnight after the PVDF membrane was blocked with 5% nonfat milk. Then the secondary antibody (HRP-conjugated goat anti-rabbit IgG, Abcam) was incubated after washing the membrane. Finally, ECL reagent (Omni-ECL™Femto Light Chemiluminescence Kit, Epizyme) was mixed and added to the PVDF membrane to avoid light. The results were visualized using an enhanced chemiluminescence western blot system G: BoxChemi X×X9 (Syngene, Cambridge, UK). Quantity One (BioRad, USA) analyzed the densities of protein bands.

### Statistical analysis

Statistical analyses were analyzed and visualized with SPSS 18.0 and GraphPad Prism. All trial data were represented as $\overset{ˉ}{X}$ ± SD. The comparisons were performed by using Student’s t-test. *P* < .05 was considered to be statistically significant.

## Results

### LncRNA ZEB1-AS1 is up-regulated in persistent *C.* *trachomatis* infected HeLa cells

**Figure 1.**
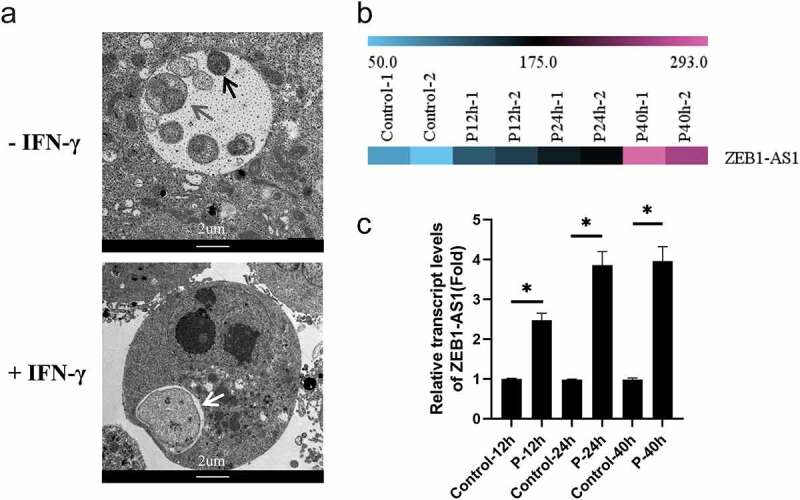
**LncRNA ZEB1-AS1 was up-regulated in HeLa cells by persistent *C. trachomatis* infection**. (a) Characterization of persistent *C. trachomatis* infection. the persistent infection group was cultured with IFN-γ (75 U/mL) after infection under the same conditions, whereas the acute infection group was not treated with IFN-γ. the inclusions in cells were mixed and detected by transmission electron microscopy; the elementary body was indicated by the black arrow, the reticulate body was indicated by gray arrow, abnormal body was indicated by white arrow, scale bar = 2um. (b) Heatmap of ZEB1-AS1 expression levels between the 12 hpi groups, the 24 hpi groups, the 40 hpi groups and the control groups in microarray data. the pink color indicates a relatively higher expression, whereas the blue indicates a relatively lower expression. (c) Validate the expression levels of ZEB1-AS1 in the microarray data using qRT-PCR. All data were presented as mean ± standard deviation (SD). *, *P* <.05.

ZEB1-AS1, a lncRNA with 2,503 bps in length, is located on chromosome 10p11.22. To identify the expression changes of ZEB1-AS1 in persistent *C. trachomatis* infection, we first constructed the persistent infection model induced by IFN-γ (75 U/mL) in HeLa cells. Compared with acute infection, abnormal bodies with a loose structure and abnormal volume were found in the inclusions of persistent infection, while elementary bodies and reticulate bodies were rare (Figure 1(a)). Then, according to our previous microarray data, we identified ZEB1-AS1 up-regulated expression in IFN-γ-induced persistent infection (Figure 1(b)). We examined its expression in persistent infection and IFN-γ control groups to validate the microarray data using qRT-PCR. As shown in Figure 1(c), the ZEB1-AS1 expression level in persistent infection group was approximately 4-fold higher than that in the IFN-γ control group at 24hpi. Up to 40hpi, its expression level did not change significantly. These results indicated that ZEB1-AS1 was up-regulated in response to persistent *C. trachomatis* infection.

### LncRNA ZEB1-AS1 is mainly located in the cytoplasm

**Figure 2.**
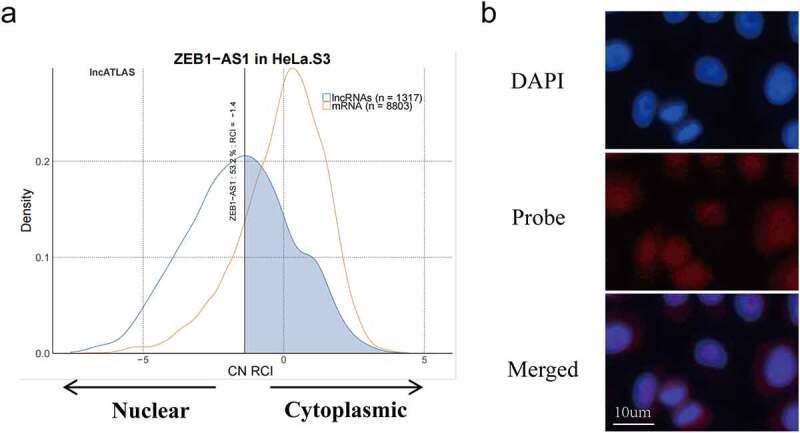
**LncRNA ZEB1-AS1 was mainly located in the cytoplasm in HeLa cells**. (a) the lncATLAS platform predicted the distribution of ZEB1-AS1 in HeLa cells. (b) the localization of ZEB1-AS1 in HeLa cells was examined by RNA-FISH. ZEB1-AS1 probes were labeled with cy3, scale bar = 10um.

It is noteworthy that lncRNAs act in different mechanisms based on their cellular location [24]. LncRNAs located in the cytoplasm may regulate gene expression at the post-transcriptional level through the competing endogenous RNAs (ceRNA) mechanism [25]. Subsequently, we found the subcellular localization of ZEB1-AS1 in lncATLAS (https://lncatlas.crg.eu/). Based on the persistently infected HeLa cell model, we selected the HeLa cells from the database and found that 53.2% of the ZEB1-AS1 was localized in the cytoplasm (Figure 2(a)). In addition, we implemented RNA FISH in HeLa cells to verify the subcellular location of ZEB1-AS1. Pictures showed that ZEB1-AS1 was mainly located in the cytoplasm, similar to the predicted results (Figure 2(b)).

### LncRNA ZEB1-AS1 regulates mitochondria-mediated host cell apoptosis

**Figure 3.**
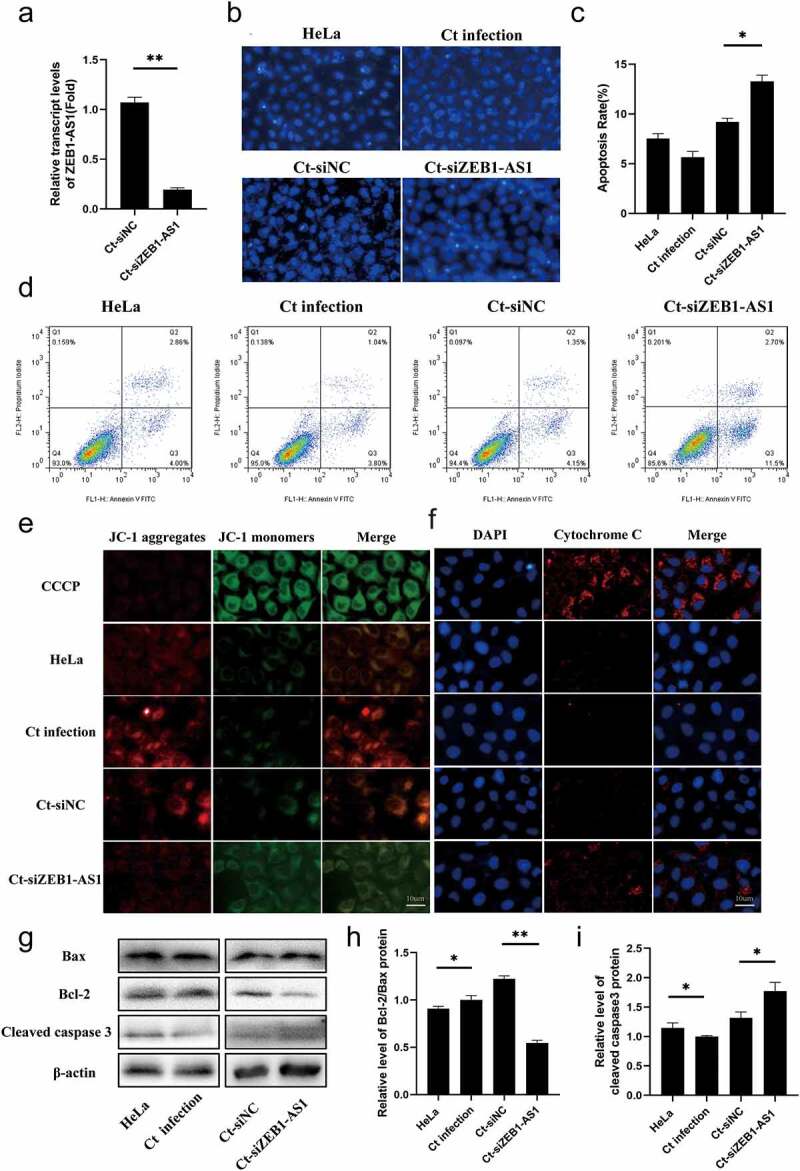
**LncRNA ZEB1-AS1 expression could affect persistent *C. trachomatis* infection-induced antiapoptosis**. (a) qRT-PCR was used to detect the effect of ZEB1-AS1 interference by siRNA. *18S rRNA* was used as the internal control. (b) the apoptotic rate was detected by Hoechst staining (400×), HeLa cells were treated with siRNA for 24 h and followed by persistent infection. (c) the apoptosis rate was calculated by the Hoechst staining experiment. (d) the apoptotic rate was detected by FCM. (e) the fluorescence intensity of JC-1 in mitochondria was observed by fluorescence microscopy; the positive control group was treated with 10 μM CCCP for 20 min before detection; HeLa cells were treated with siZEB1-AS1 for 24 h and then were followed persistent infection, scale bar = 10um. (f) Cytochrome c fluorescent intensity per cell was assayed by fluorescence microscope, scale bar = 10um. (g, h, i) the indicated protein levels of Bax, Bcl-2, and cleaved caspase 3 were analyzed by western blot. All data were presented as mean ± standard deviation (SD), *, *P* <.05, **, *P* <.01.

C.*trachomatis* could proliferate itself by resisting host cell apoptosis, one self-protection mechanisms [26]. However, many underlying molecular mechanisms for host manipulation remain poorly understood. Current evidence suggests that ZEB1-AS1 was associated with apoptosis and tumor proliferation [27,28], thus we further discussed the role of ZEB1-AS1 in the process of anti-host cell apoptosis during persistent infection. Up-regulated ZEB1-AS1 was inhibited by specific siRNA, which was significantly down-regulated compared with Ct-siNC (*P* < .01) (Figure 3(a)). Hoechst staining was used to detect the number of apoptotic bodies to determine the antiapoptosis status of each group. The results revealed that the apoptosis rate of the Ct-siZEB1-AS1 group was 13.27%, which was higher than that of the Ct-siNC control group (9.21%) (Figure 3(b–c), *P* < .05). Afterward, flow cytometry further verified the apoptotic effect of siZEB1-AS1, and the results were consistent with Hoechst staining (Figure 3(d)).

Mitochondria play a central role in the life and death of cells [29]. They produce the energy required for survival and participate in the intrinsic pathway regulating apoptosis [30]. Decreased mitochondrial membrane potential (MMP) is one of the crucial factors leading to apoptosis and is considered the first step of the apoptosis cascade [31]. To further investigate whether ZEB1-AS1 affected mitochondrial permeability by changing MMP, we used JC-1 fluorescent probe to detect MMP. CCCP (10 µM) was used as a positive control to induce MMP decline. Our results showed that the MMP of the Ct-siZEB1-AS1 cells was significantly lower than that in the Ct-siNC cells. The ratio of JC-1 aggregates/monomers decreased if ZEB1-AS1 was silenced (Figure 3(e)). In addition, considering that the change of mitochondrial permeability could lead to the release of cytochrome c to promote cell apoptosis, we detected the release of cytochrome c by immunofluorescence, and the pictures showed that the content of cytochrome c in the cytoplasm of the Ct-siZEB1-AS1 group was significantly increased (Figure 3(f)).

Furthermore, we tested the classical apoptosis-related molecules in the Ct-siZEB1-AS1 group, such as cleaved caspase 3, Bax, and Bcl-2 (Figure 3(g)). Compared with the Ct-siNC group, the Bcl-2/Bax ratio of the Ct-siZEB1-AS1 group was down-regulated (Figure 3(h), *P* < .01), and the percentage of cleaved caspase 3 to internal reference was increased (Figure 3(i), *P* < .05). Hence, we identified ZEB1-AS1 might play a role in persistent *C. trachomatis* infection-induced antiapoptosis.

### LncRNA ZEB1-AS1 serves as a ceRNA for miR-1224-5p to up-regulate MAP4K4 in persistent *C.* *trachomatis* infection

**Figure 4.**
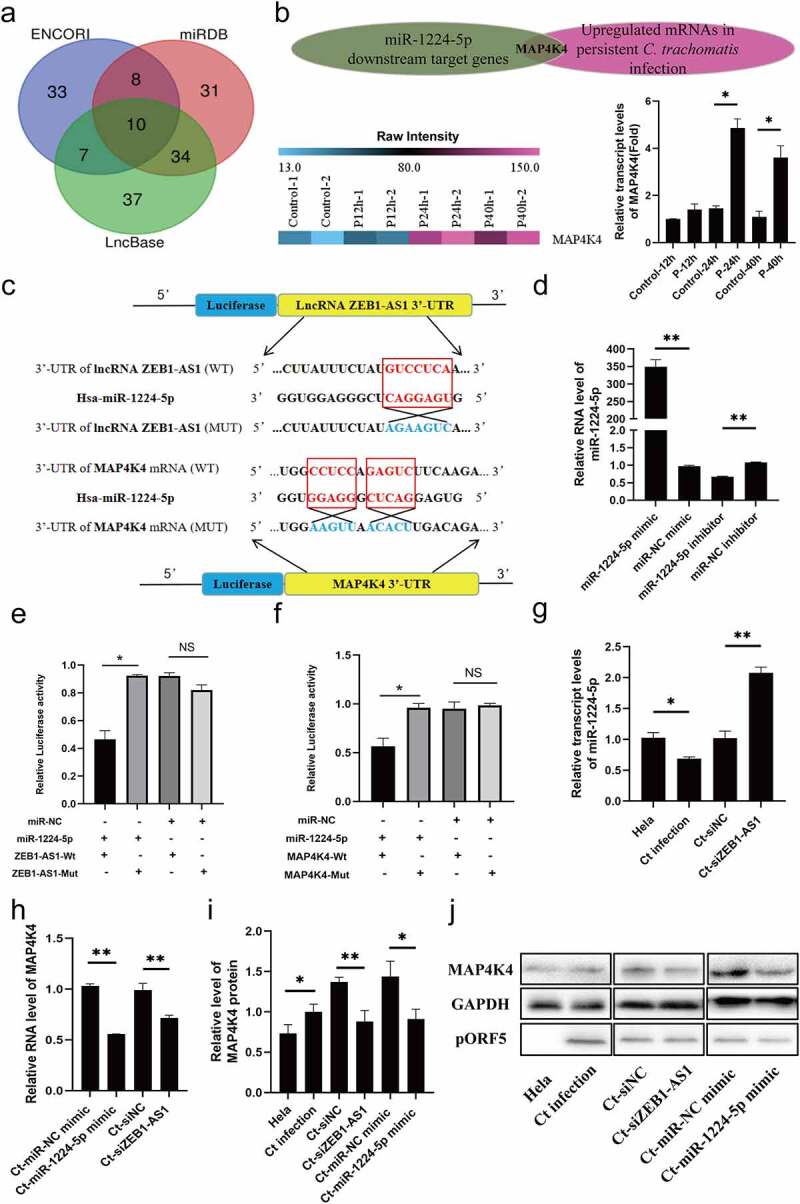
**miR-1224-5p was a target of lncRNA ZEB1-AS1, and MAP4K4 was a direct target gene of miR-1224-5p in persistent *C. trachomatis* infected HeLa cells**. (a) the Venn diagram showed that Lncbase, ENCORI, and miRDB platforms predicted lncRNA-targeted miRnas. (b) Heatmap of MAP4K4 expression levels between the 12 hpi groups, the 24 hpi groups, the 40 hpi groups, and the control groups in microarray data. the pink color indicates a relatively higher expression, whereas the blue indicates a relatively lower expression. Validate the expression levels of MAP4K4 in the microarray data using qRT-PCR. All data were representative of three independent experiments. (c) the binding sites of miR-1224-5p and ZEB1-AS1 or MAP4K4. (d) qRT-PCR was used to validate the effect of miR-1224-5p expression by miR-1224-5p mimic or inhibitor. U6 was used as the internal control. (e) the luciferase reporter gene assay verified the correlation between miR-1224-5p and ZEB1-AS1. (f) the luciferase reporter gene assay confirmed the correlation between miR-1224-5p and MAP4K4. (g) the expression of miR-1224-5p was detected in the HeLa group, persistent infection group, Ct-siNC group, and Ct-siZEB1-AS1 group by qRT-PCR. (h) the expression of MAP4K4 was detected in Ct-miR-NC mimic group, Ct-miR-1224-5p mimic group, Ct-siNC group, and Ct-siZEB1-AS1 group by qRT-PCR. (i, j) Western blot assays were conducted to explore the effects of ZEB1-AS1 and miR-1224-5p in MAP4K4 expression in persistent infection cells. All data were presented as mean ± standard deviation (SD), *, *P* <.05, **, *P* <.01, NS, no significance.

Based on the cellular localization of ZEB1-AS1, we began to explore its potential molecular mechanism. By using Lncbase (http://carolina.imis.athena-innovation.gr/diana_tools/web/index.php?r=lncbasev2/index), ENCORI (http://starbase.sysu.edu.cn/) and miRDB (http://mirdb.org/) websites, a total of 160 underlying miRNAs interacting with ZEB1-AS1 were screened out. The overlapping part revealed ten common miRNAs (Figure 4(a)). Following literature analysis, we found that miR-200a-3p and miR-1224-5p were involved in regulating cell apoptosis [32–35]. In addition, we predicted the expression abundance of miRNA based on the Tissuealtas (https://ccb-web.cs.uni-saarland.de/tissueatlas/). The expression level of miR-1224-5p in uterus tissue was higher than that of miR-200a-3p. Subsequently, we found that miR-1224-5p showed a lower expression in the persistent *C. trachomatis* infection group compared with the uninfected group using qRT-PCR (Figure 4(g), *P* < .05). Thus, miR-1224-5p was finally selected as the target molecule of ZEB1-AS1 in persistent *C. trachomatis* infection.

Similarly, we screened mitogen-activated protein 4 kinase 4 (MAP4K4) by taking the intersection of the miR-1224-5p downstream target genes predicted by the ENCORI and the up-regulated differential mRNAs based on the persistent infection chip database. Subsequently, we verified the expression of MAP4K4 in the microarray using qRT-PCR and was normalized by internal control *18S rRNA* expression. The results were similar to the trends observed in microarray data (Figure 4(b), *P* < .05). As a STE20/MAP4K family member, MAP4K4 played a critical regulatory role in cellular signal transduction, including cell cycle, apoptosis, and cell growth [36–38]. Thus, we began to focus on MAP4K4 for the following study. The RNA binding prediction sites of miR-1224-5p, ZEB1-AS1, and MAP4K4 are shown in Figure 4(c). We constructed WT and MUT vectors to verify the interaction sites of miR-1224-5p and ZEB1-AS1 and MAP4K4 by the dual-luciferase reporter gene. First, the transfection expression levels of miR-1224-5p mimic (50 nM) and miR-1224-5p inhibitor (200 nM) were verified by qRT-PCR (Figure 4(d), *P* < .01). Then, we found that high expression of miR-1224-5p attenuated the luciferase activity in the ZEB1-AS1 WT group and MAP4K4 WT group. But, there was no difference in the ZEB1-AS1 MUT group and MAP4K4 MUT group (Figure 4(e–f), *P* < .05). Interestingly, when ZEB1-AS1 was silenced, the expression of miR-1224-5p was significantly increased (Figure 4(g), *P* < .01). We indicated that ZEB1-AS1 acted as a sponge for miR-1224-5p in persistently infected HeLa cells. Moreover, we found that the expression of MAP4K4 was significantly decreased after transfection of miR-1224-5p mimic (Figure 4(h), *P* < .01). These results indicated that miR-1224-5p directly targeted MAP4K4 in persistently infected HeLa cells.

Based on these results, we analyzed whether ZEB1-AS1 regulates the expression of MAP4K4 at the RNA and protein levels. In persistent *C. trachomatis* infected HeLa cells, silencing ZEB1-AS1 repressed the expression of MAP4K4 (Figure 4(h–j), *P* < .01). In addition, miR-1224-5p overexpression attenuated the expression of MAP4K4 (Figure 4(h–j), *P* < .05). These data proved that ZEB1-AS1 indirectly regulated the expression of MAP4K4 through sponge miR-1224-5p.

### LncRNA ZEB1-AS1 hinders mitochondria-mediated host cell apoptosis via regulating the miR-1224-5p/MAP4K4 axis

**Figure 5.**
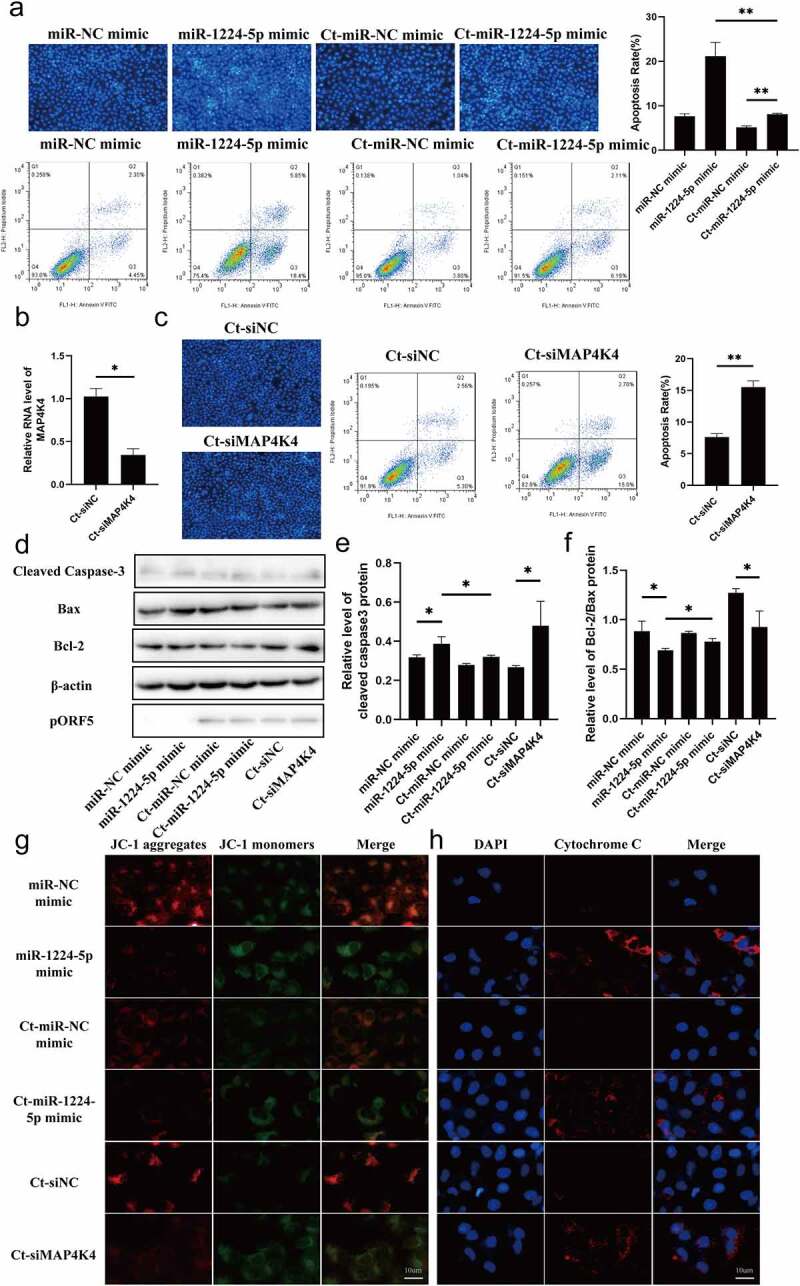
**miR-1224-5p and MAP4K4 were involved in the antiapoptosis of persistent *C. trachomatis* infection**. (a) the apoptotic rate was detected by Hoechst staining (200×) and FCM, HeLa cells were transfected with miR-1224-5p mimic or miR-NC mimic for 24 h and then were followed persistent infection. the apoptosis rate was calculated by the Hoechst staining experiment. (b) qRT-PCR was used to validate the effect of MAP4K4 expression by siRNA. *18S rRNA* was used as the internal control. (c) the apoptotic rate was detected by Hoechst staining (200×) and FCM, HeLa cells were transfected with siMAP4K4 or siNC for 24 h and then were followed persistent infection. the apoptosis rate was calculated by the Hoechst staining experiment. (d, e, f) the indicated protein levels of Bax, Bcl-2, and cleaved caspase 3 were analyzed by western blot, the gray values of protein bands were quantified by Image J. (g) the fluorescence intensity of JC-1 in mitochondria was observed by fluorescence microscopy, HeLa cells were treated with miR-1224-5p mimic or siMAP4K4 for 24 h and then were followed persistent infection, scale bar = 10um. (h) Cytochrome c fluorescent intensity per cell was assayed by fluorescence microscope, scale bar = 10um. All data were presented as mean ± standard deviation (SD), *, *P* <.05, **, *P* <.01.

To investigate whether miR-1224-5p/MAP4K4 axis is involved in regulating ZEB1-AS1 on persistent *C. trachomatis* infection-induced antiapoptosis, we focused on the functional experiments of the miR-1224-5p/MAP4K4 axis. Hoechst staining showed that miR-1224-5p overexpression elevated the apoptosis rate of HeLa cells, which was alleviated by the persistent *C. trachomatis* infection (Figure 5(a), *P* < .01). Flow cytometry analyses were consistent with Hoechst’s staining results (Figure 5(a)). To verify the effect of MAP4K4 on persistent *C. trachomatis* infection-induced antiapoptosis, we used siRNA to silence MAP4K4 expression (Figure 5(b), *P* < .05). The Hoechst staining and flow cytometry results showed that siMAP4K4 significantly promoted apoptosis of infected cells (Figure 5(c), *P* < .01). Western blot results also revealed that miR-1224-5p mimic and Ct-siMAP4K4 groups reduced the Bcl-2/Bax ratio and increased cleaved caspase 3 expression compared with the negative control group at protein level (Figure 5(d–f), *P* < .05). Similarly, miR-1224-5p mimic and siMAP4K4 caused the suppression of MMP (Figure 5(g), *P* < .05) and increased the release of cytochrome c (Figure 5(h), *P* < .05), which could be alleviated by *C. trachomatis* infection. These results suggested that the ZEB1-AS1/miR-1224-5p/MAP4K4 axis regulated mitochondria-mediated host cell apoptosis in persistent *C. trachomatis* infection.

### LncRNA ZEB1-AS1 augments persistent *C.* *trachomatis* infection-induced antiapoptosis via MAPK/ERK signaling pathway

**Figure 6.**
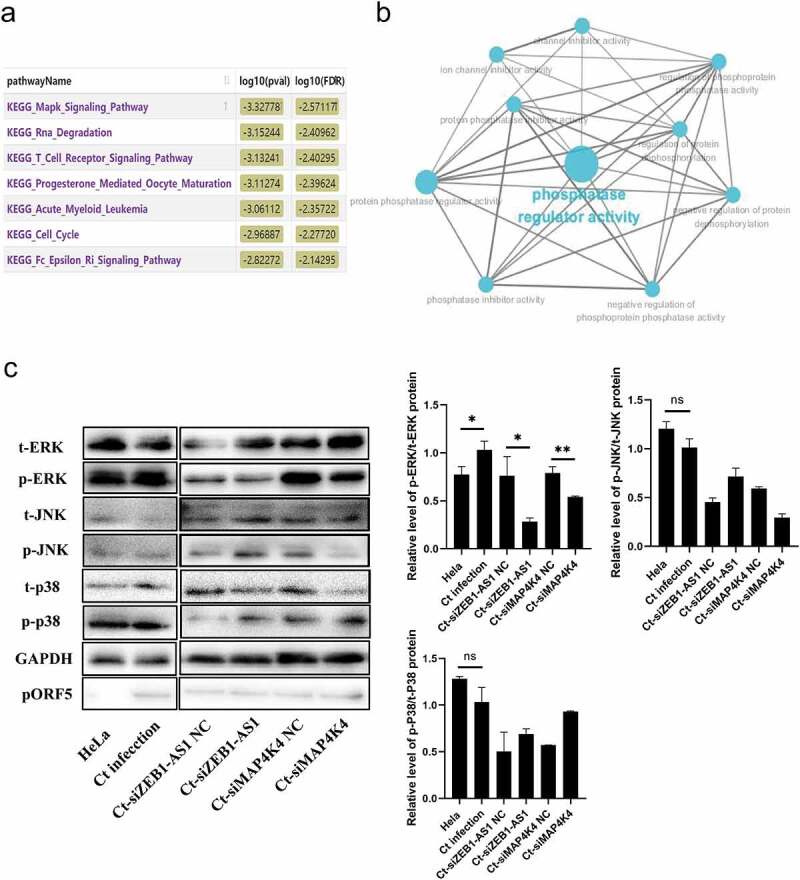
**LncRNA ZEB1-AS1 and MAP4K4 activated the MAPK/ERK pathway in persistent *C. trachomatis* infection**. (a) Bioinformatics analysis of the enrichment pathway of ZEB1-AS1 co-expressed mRnas. (b) GO enrichment of ZEB1-AS1-targeted mRnas. the blue dots represent related biological processes. (c) the p-ERK, t-ERK, p-JNK, t-JNK, p-p38, and t-p38 protein expressions were evaluated using western blot in persistent infection cells with different transfection molecules. the gray values of protein bands were quantified by Image J. All data were presented as mean ± standard deviation (SD), *, *P* <.05, NS, no significance.

Previous data confirmed that ZEB1-AS1 could regulate mRNA MAP4K4 expression. Next, we constructed a co-expression network of ZEB1-AS1 and differentially expressed mRNAs and analyzed the enrichment pathway of ZEB1-AS1 co-expressed molecules by Co-lncRNA. As shown in Figure 6(a), ZEB1-AS1 co-expressed molecules were mainly concentrated in the “mitogen-activated protein kinase (MAPK) signaling pathway,” “RNA degradation,” “T cell receptor signaling pathway,” and so on. Furthermore, ClueGo Plugin analyzed the ZEB1-AS1 targeted mRNAs. Interestingly, we found that they were involved in phosphatase regulator activity (Figure 6(b)). Based on the above bioinformatics analysis and our previous work [39], we further explored the regulatory pattern between ZEB1-AS1 and MAPK signals in persistent *C. trachomatis* infection. Among MAPK signaling pathways, coincidentally, MAP4K4 plays a critical regulatory role [40]. There are three prominent mammalian MAPK: extracellular signal-regulated kinase (ERK), c-Jun N-terminal kinase (JNK), and p38 kinase [41]. Therefore, we implemented phosphorylation levels of ERK, JNK, and p38 experiments in the ZEB1-AS1 and MAP4K4 silencing groups. Our results revealed that the persistent *C. trachomatis* infection group augmented the level of p/t-ERK compared to the IFN-γ control group. Still, the levels of p/t-JNK and p/t-p38 were not statistically significant (Figure 6(c), *P* < .05), indicating the persistent *C. trachomatis* infection-mediated activation of the MAPK/ERK signal. Silence of ZEB1-AS1 or MAP4K4 remarkably decreased the levels of p/t-ERK in the persistent infection group, indicating that silenced ZEB1-AS1 or MAP4K4 could reverse the activation of MAPK/ERK signal in persistent *C. trachomatis* infection (Figure 6(c), *P* < .05).

## Discussion

This work identified a key lncRNA ZEB1-AS1, a persistent *C. trachomatis* infection up-regulated lncRNA, that could enhance persistent infection-induced antiapoptosis. Given the location of ZEB1-AS1, we speculated that it might negatively regulate miRNA functions or participate in the ceRNA regulatory network. Therefore, we focused on miR-1224-5p through the prediction and functional analysis of ZEB1-AS1 binding molecules. To date, it has been reported that miR-1224-5p was involved in promoting apoptosis of keloid fibroblast [33], suggesting that it may mediate the fate of cells with persistent *C. trachomatis* infection.

Inhibition of host cell apoptosis is a persistent *C. trachomatis* infection mechanisms [42]. Current studies have confirmed that persistent *C. trachomatis* infection affects some apoptotic signaling pathways [43–45]. Researchers found that the p53-MDM2 axis interfered with the chlamydial antiapoptosis effect on host cells [46]. Besides, the PI3K/Akt signaling pathway has also been identified in antiapoptosis specifically triggered by *C. pneumoniae* in neutrophils [47]. In viral infections by Herpes, two small RNAs of the herpes simplex virus type 1 latency-associated transcript play a role in the latency-reactivation cycle by inhibiting apoptosis and productive infection [48]. Another virally encoded RNA (beta2.7) interacted with mitochondrial enzyme complex I to resist host cell apoptosis signals [49]. This interaction is significant for stabilizing the MMP resulting in continued ATP, which is critical for the virus’ life cycle. In the present study, we found that the ZEB1-AS1/miR-1224-5p/MAP4K4 axis regulated persistent *C. trachomatis* infection-induced antiapoptosis in a mitochondria-dependent manner. Here we note that besides being well studied in mitochondrial-mediated apoptosis, the Bcl-2 family also has non-apoptotic effects, including mitochondrial division and mitochondrial respiration [50]. Yusuke Kurihara et al. indicated that mitochondrial stretching and division occurred in the early and late stages of *C. trachomatis* infection, respectively [51]. These provide new insights into the persistent survival of *C. trachomatis*.

The transcriptomic profiles in persistent *C. trachomatis* infection have been determined. The up-regulated ZEB1-AS1 was proposed to be involved in the MAPK signaling pathway by the lncRNA-mRNA co-expression network. We predicted the putative target genes of miR-1224-5p using ENCORI. MAP4K4 was chosen for our focus, which belongs to the STE20/MAP4K family and has been reported to be involved in cell apoptosis [52–54]. Previous studies have shown that the antiapoptosis activity of *Chlamydia* requires activation of the MAPK/ERK survival pathway [39,55,56]. And Liu Y et al. identified non-coding circular RNAs that are involved in the regulation MAPK signaling pathway during the process of *Chlamydia* infection [57]. Our research group has also found that *Chlamydia* induces autophagy by activating unfolded protein response through MAPK/ERK signaling pathway [58]. In particular, *Chlamydia*-induced inflammatory cytokines are also associated with starting the MAPK signaling pathway [59]. Interestingly, the upregulation of glucose transporter 1 (GLUT1) and glucose transporter 3 (GLUT3) in mammalian cells during *Chlamydia* infection is dependent on *Chlamydia*-induced MAPK kinase activation [60], suggesting that *Chlamydia* survival depends on the MAPK signaling pathway. Noticeably, MAP4K4 can induce ERK, JNK, and p38 activation in various pathological environments [40,61]. In this work, we found a decrease of ZEB1-AS1 or MAP4K4 down-regulated persistent *C. trachomatis* infection-induced elevation of p-ERK level. These results characterize the role of ZEB1-AS1/miR-1224-5p/MAP4K4 molecular axis in apoptosis resistance induced by MAPK/ERK pathway activation in persistent *C. trachomatis* infection. It also provides us with exciting research directions. Targeting the ZEB1-AS1/miR-1224-5p/MAP4K4 axis may provide new insights into understanding the molecular mechanisms of persistent *C. trachomatis* infection and reveal a potential therapeutic strategy for its treatment.

## Supplementary Material

Supplemental Material

## Data Availability

The data that support the findings of this study are available from the corresponding author upon reasonable request.
