# Supplementary material for: LncRNA ZEB1-AS1/miR-1224-5p / MAP4K4 axis regulates mitochondria-mediated HeLa cell apoptosis in persistent Chlamydia trachomatis infection
Source: Virulence. 2022 Mar 10;13(1):444–57. doi: 10.1080/21505594.2022.2044666 (PMC8920228; doi:10.1080/21505594.2022.2044666)
Supplement: Supplemental Material [file KVIR_A_2044666_SM3274.docx]

**Supplementary Table 1. Sequences used for qRT-PCR, siRNA and probes in this study**

| Primers | siRNA/Probe |
| --- | --- |
| ZEB1-AS1 (product size: 156)  Forward: TGCTTGTCTCACTTCCCCAT  Reverse: AGGAATTCATGGCCTGTGGA | siZEB1-AS1  Forward: GAAAGAGAGGCUAGAAGUUCC  Reverse: AACUUCUAGCCUCUCUUUCAA |
| miR‐1224‐5p  Forward: GTGAGGACTCGGGAGGTGG | siZEB1-AS1 NC  Forward: UUCUCCGAACGUGUCACGUTT  Reverse: ACGUGACACGUUCGGAGAATT |
| MAP4K4 (product size: 180)  Forward: AGTGGGAGAAGGCAGAGAAC  Reverse: AGCTCAGACCCCTTTACCAC | siMAP4K4  Forward: GAACAAAGGAUGUGGCAUATT  Reverse: UAUGCCACAUCCUUUGUUCTT |
| 18S rRNA (product size: 116)  Forward: CGCTCGCTCCTCTCCTACTT  Reverse: CGGGTTGGTTTTGATCTGATAA | siMAP4K4 NC  Forward: UUCUCCGAACGUGUCACGUTT  Reverse: ACGUGACACGUUCGGAGAATT |
| U6 (product size: 87)  Forward: CGCTTCGGCAGCACATATAC  Reverse: TTCACGAATTTGCGTGTCATC | miR-1224-5p mimic  Forward: GUGAGGACUCGGGAGGUGG  Reverse: CCACCUCCCGAGUCCUCAC |
|  | miR-1224-5p mimic NC  Forward:UCACAACCUCCUAGAAAGAGUAGA  Reverse:UCUACUCUUUCUAGGAGGUUGUGA |
|  | miR-1224-5p inhibitor  CCACCUCCCGAGUCCUCAC |
|  | miR-1224-5p inhibitor NC  UCUACUCUUUCUAGGAGGUUGUGA |
|  | ZEB1-AS1 probe-1  CGTGTGGGTATTACTCATCC |
|  | ZEB1-AS1 probe-2  GGCCCAAACTAACTAAACCA |

**Supplementary Table 2. Sequences used for dual-luciferase reporter gene in this study**

| gene | Sequences |
| --- | --- |
| ZEB1-AS1 (WT) | ctcgagATGAAGTGAAGAGACAAACAGAAGTCATTTTCTTCCTTACTTTAGTGGTTTCTGGTTTAGTTAGTTTGGGCCAAACTGTGGACAAGTACCTTTTCAGGTAACTTTTTTTTCTTATTTCTATGTCCTCAACACCTAGTGGAGTACGTAGCCAATAGTAGATGCTTAATAAACATTTCTTAAATTAATATTGTTGACCTTTTCTGACCCTGTTCTTGACAGTAAGGTACATAATCTGCCTTCgcggccgc |
| ZEB1-AS1 (MUT) | ctcgagATGAAGTGAAGAGACAAACAGAAGTCATTTTCTTCCTTACTTTAGTGGTTTCTGGTTTAGTTAGTTTGGGCCAAACTGTGGACAAGTACCTTTTCAGGTAACTTTTTTTTCTTATTTCTATAGAAGTCACACCTAGTGGAGTACGTAGCCAATAGTAGATGCTTAATAAACATTTCTTAAATTAATATTGTTGACCTTTTCTGACCCTGTTCTTGACAGTAAGGTACATAATCTGCCTTCgcggccgc |
| MAP4K4 (WT) | ctcgagACAAGGTGTTCTTTGCCTCTGTTCGGTCTGGTGGCAGCAGTCAGGTTTATTTCATGACCTTAGGCAGGACTTCTCTTCTGAGCTGGTAGAAGCAGTGTGATCCAGGGATTACTGGCCTCCAGAGTCTTCAAGATCCTGAGAACTTGGAATTCCTTGTAACTGGAGCTCGGAGCTGCACCGAGGGCAACCAGGACAGCTGTGTGTGCAGACCTCATGTGTTGGGTTCTCTCCCCTCCTTCCTgcggccgc |
| MAP4K4 (MUT) | ctcgagACAAGGTGTTCTTTGCCTCTGTTCGGTCTGGTGGCAGCAGTCAGGTTTATTTCATGACCTTAGGCAGGACTTCTCTTCTGAGCTGGTAGAAGCAGTGTGATCCAGGGATTACTGGAAGTTAACACTTGACAGATCCTGAGAACTTGGAATTCCTTGTAACTGGAGCTCGGAGCTGCACCGAGGGCAACCAGGACAGCTGTGTGTGCAGACCTCATGTGTTGGGTTCTCTCCCCTCCTTCCTgcggccgc |
